# Characterization of gastric adenocarcinoma cell lines established from CEA424/*SV40 T antigen*-transgenic mice with or without a human *CEA *transgene

**DOI:** 10.1186/1471-2407-6-57

**Published:** 2006-03-14

**Authors:** Jessica Nöckel, Natasja K van den Engel, Hauke Winter, Rudolf A Hatz, Wolfgang Zimmermann, Robert Kammerer

**Affiliations:** 1 Tumor Immunology Laboratory, LIFE-Center, Klinikum Grosshadern, Ludwig-Maximilians-University, Marchioninistrasse 23, 81377 Munich, Germany; 2 Department of Surgery, Klinikum Grosshadern, Ludwig-Maximilians-University, 81377 Munich, Germany; 3 Institute for Molecular Immunology, GSF National Research Center for the Environment and Health, 81377 Munich, Germany

## Abstract

**Background:**

Gastric carcinoma is one of the most frequent cancers worldwide. Patients with gastric cancer at an advanced disease stage have a poor prognosis, due to the limited efficacy of available therapies. Therefore, the development of new therapies, like immunotherapy for the treatment of gastric cancer is of utmost importance. Since the usability of existing preclinical models for the evaluation of immunotherapies for gastric adenocarcinomas is limited, the goal of the present study was to establish murine *in vivo *models which allow the stepwise improvement of immunotherapies for gastric cancer.

**Methods:**

Since no murine gastric adenocarcinoma cell lines are available we established four cell lines (424GC, mGC3, mGC5, mGC8) from spontaneously developing tumors of CEA424/SV40 T antigen (CEA424/Tag) mice and three cell lines derived from double-transgenic offsprings of CEA424/Tag mice mated with human carcinoembryonic antigen (CEA)-transgenic (CEA424/Tag-CEA) mice (mGC2^CEA^, mGC4^CEA^, mGC11^CEA^). CEA424/Tag is a transgenic C57BL/6 mouse strain harboring the Tag under the control of a -424/-8 bp CEA gene promoter which leads to the development of invasive adenocarcinoma in the glandular stomach. Tumor cell lines established from CEA424/Tag-CEA mice express the well defined tumor antigen CEA under the control of its natural regulatory elements.

**Results:**

The epithelial origin of the tumor cells was proven by morphological criteria including the presence of mucin within the cells and the expression of the cell adhesion molecules EpCAM and CEACAM1. All cell lines consistently express the transgenes CEA and/or Tag and MHC class I molecules leading to their susceptibility to lysis by Tag-specific CTL *in vitro*. Despite the presentation of CTL-epitopes derived from the transgene products the tumor cell lines were tumorigenic when grafted into C57BL/6, CEA424/Tag or CEA424/Tag-CEA-transgenic hosts and no significant differences in tumor take and tumor growth were observed in the different hosts. Although no spontaneous tumor rejection was observed, vaccination of C57BL/6 mice with lysates from gastric carcinoma cell lines protected C57BL/6 mice from tumor challenge, demonstrating the tumorigenicity of the tumor cell lines in nontransgenic mice of the H-2^b ^haplotype.

**Conclusion:**

These tumor cell lines grafted in different syngeneic hosts should prove to be very useful to optimize immunotherapy regimens to be finally tested in transgenic animals developing primary gastric carcinomas.

## Background

Gastric cancer is the second most common cancer world-wide[1]. It is often not detected until an advanced stage; consequently, the 5-year survival rates are low (10 to 20%). Owing to local invasion and metastasis, radiation therapy or chemotherapy does not significantly increase the length or quality of life of patients with advanced gastric cancer. Therefore, development of new neoadjuvant and adjuvant treatment modalities are needed. Immunotherapy might be a promising alternative option. A number of immunotherapy approaches like adoptive transfer of tumor-specific T cells, and vaccination using either undefined tumor antigens derived from tumor lysates and tumor cell lines or defined tumor antigens commonly presented by dendritic cells are being evaluated for various cancers[2,3]. For gastric cancers, immunotherapy was not taken seriously into consideration due to the concept that gastric cancer is poorly immunogenic. Therefore, only a small number of clinical immunotherapy trials have been reported [4-7]. In addition, only a limited number of tumor-associated antigens with potential use for immunotherapy have been identified [8-11]. Consequently, the capability of the immune system to recognize and eradicate gastric cancers is largely unknown. To gain insight into the efficacy of various immunotherapies for the treatment of gastric cancer and to elucidate the underlying mechanism of induced immune responses animal models of gastric adenocarcinoma are indispensable.

To this end, several groups including ours have recently established transgenic or knock-out mouse strains which develop gastric adenomas or adenocarcinomas in various parts of the stomach after different latencies[12]. We have developed a transgenic gastric carcinoma C57BL/6 mouse model based on a SV40 large T antigen (SV40 Tag) transgene controlled by a human carcinoembryonic antigen (CEA) gene promoter (from -424 to -8 of the translational start site)[13]. In 100% of the animals, dysplastic crypt formation in the stomach mucosa is observed in the pyloric region already in 30 day old CEA424/SV40 Tag-transgenic mice. Dysplasia progresses to invasive carcinomas and by day 50 the whole pyloric gastric mucosa has been replaced by carcinoma cells. Between the age of 90 to 110 days the transgenic mice become moribund and die probably of undernourishment due to blockage of the pylorus[13]. The control of Tag expression by a minimal CEA gene promoter allows the tumor-directed expression of CEA by crossing CEA424/SV40 Tag-transgenic mice with human *CEA*-transgenic C57BL/6 mice, which express the CEA transgene in a similar spatiotemporal expression pattern as found in humans[13,14]. The human tumor marker CEA is expressed in many human adenocarcinomas including more than 50% of gastric carcinomas[15,16]. CEA is increasingly used as target antigen for a variety of antibody- and cell-mediated tumor immunotherapy approaches [17-19]. CEA and Tag are suitable immunotherapy target antigens since a number of T cell epitopes of these antigens have been identified in C57BL/6 mice [20-22]. Although these transgenic mouse strains mirror very closely gastric adenocarcinoma development in humans, experimentation with these mice is relatively time consuming and expensive due to the difficulties to determine tumor growth and the requirement of breeding transgenic mice. Therefore, it is desirable to have a syngeneic transplantable tumor system of gastric adenocarcinomas in immunocompetent mice for optimization of a given immunotherapy protocol before it is evaluated in the transgenic mice. Here we describe murine gastric adenocarcinoma cell lines established from spontaneously developing tumors of SV40 Tag-transgenic mice that are tumorigenic in both syngeneic wild type and transgenic mice.

## Methods

### Mouse strains and cell lines

CEA424/Tag-transgenic mice (C57BL/6-Tg(CEACAM5-Tag)L5496Wzm), CEA-transgenic mice (C57BL/6-Tg(CEACAM5)2682Wzm; CEA2682) and F1 mice from a cross between CEA424/Tag-transgenic and CEA-transgenic mice have been described previously [13,14]. Meanwhile the transgenic lines have been backcrossed to C57BL/6 mice (H-2^b^) for more than 15 generations. The transgenic lines as well as C57BL/6 mice (Charles River, Sulzfeld, Germany) were bred and kept under standard pathogen-free conditions in the animal facility of the Institute for Surgical Research, Ludwig-Maximilians-University of Munich. The animal experiments were performed after approval by the local animal welfare committee. For tumorigenicity and immunogenicity assays mice were used at 8–12 weeks of age. African green monkey kidney Cos7L cell line was obtained from the American Tissue Culture Collection (ATCC, Rockville, MD). The BALB/c-derived fibrosarcoma Meth-A was kindly provided by W. Deppert (Heinrich-Pette-Institut, Hamburg). Meth-A-CEA cells were obtained by transfection of Meth-A cells with the pRc/CMV-CEA expression plasmids using FuGENE™ 6 transfection reagent (Roche Molecular Biochemicals, Switzerland) according to the manufacturer's instruction. Meth-A-cTag and RBL5/T transfectants were previously described[23].

### Establishment of gastric carcinoma cell lines

The gastric carcinomas used to establish tumor cell lines were obtained from 8 different, 13 week-old mice. Four derived from CEA424/Tag-transgenic mice and 4 from CEA424/TAg-CEA-double transgenic mice. The names of the cell lines derived from the latter mice are marked by the superscript "CEA". All cultures were performed in RPMI1640 supplemented with 10% heat inactivated fetal calf serum (FCS "Gold"; PAA Laboratories, Coelbe, Germany), 2 mM L-glutamine, 100 U/ml penicillin, 100 μg/ml streptomycin, non-essential amino acids and 1 mM sodium pyruvate (GIBCO/Invitrogen, Karlsruhe, Germany), further referred to as tumor medium (TM). Tumor tissues were extensively washed in phosphate-buffered saline (PBS) supplemented with 200 μg/ml gentamicin and 2.5 μg/ml amphotericin B (GIBCO/Invitrogen), cut into 1 mm^3 ^pieces with a scalpel and plated in tissue culture flasks containing TM. The culture medium was changed every 3–4 days. Epithelial cells and fibroblasts growing out of the tissue fragments were separated by cell scraping, selective trypsinization and selective passaging with 1,000 U/ml collagenase and 500 U/ml hyaluronidase (Biochrom, Berlin, Germany). During dissociation, the flasks were monitored under an inverted microscope and digestion was stopped when fibroblasts but not epithelial cells were detached (trypsin) or vice versa (collagenase/hyaluronidase). This procedure was repeated weekly until all fibroblasts were eliminated from the tumor cell cultures. During generation of the 424GC cell line, a fibroblast culture was established from contaminating fibroblasts (424 fibroblasts). Spheroids formed within 5–7 days after seeding 0.5 × 10^3 ^mGC8 cells into Noble agar (Sigma-Aldrich, Taufkirchen, Germany)-coated 96-well plates (TPP-Biochrom, Berlin, Germany) in 200 μl TM which was replaced with fresh medium every two days. To assess viability of cells on the surface of the spheroids, spheroids were incubated with FITC-labeled annexin V (Annexin V FITC Apoptosis Detection Kit; Calbiochem, Merck Biosciences, Darmstadt, Germany) for detection of apoptotic cells or with propidium iodide to identify necrotic cells according to the manufacturer's recommendations.

### Flow cytometry analyses

For surface staining cells were trypsinized, washed with PBS and suspended in PBS/0.5% w/v bovine serum albumin (BSA) supplemented with 0.02% w/v sodium azide. For induction of MHC molecules, cells were incubated with 20 ng/ml of interferon-γ (IFNγ ; Peprotec, London, UK) for 24 hours prior harvesting. Non-specific binding of antibodies to Fc receptors was blocked by preincubation of the cells with 1 μg/10^6 ^cells of anti-CD16/CD32 monoclonal antibody (mAb) 2.4G2 (BD Pharmingen, Heidelberg, Germany) for 15 min. Subsequently the cells were incubated with 0.5 μg/10^6 ^cells of the mAb of interest for 30 min at 4°C, washed twice, and where appropriate, subsequently reacted with a second-step antibody for 15 min at 4°C. Cells were washed twice and analyzed using a FACScan (BD, Mountain View, CA). Dead cells were excluded by propidium iodide staining. The following reagents and mAbs against murine antigens from BD Pharmingen were used: phycoerythrin (PE)-conjugated mouse IgG_2a _anti-I-A^b^, biotinylated mouse IgG_2a _anti-H-2D^b^, PE-conjugated mouse IgG_2a_anti-H-2K^b^, PE-conjugated anti-mouse CD80/B7-1, PE-conjugated rat IgG_2a _anti-mouse CD40, PE-conjugated rat IgG_2a _anti-mouse CD86/B7-2, fluorescein isothiocyanate (FITC)-conjugated, Armenian hamster IgG_2 _anti-mouse CD80/B7-1. FITC-conjugated rat IgG_1 _mAb R3-34, PE-conjugated rat IgG_1 _mAb R3-34, PE-conjugated mouse IgG_2a _anti-rat SIRP, FITC-conjugated Armenian hamster IgG_2_anti-KLH and PE-conjugated rat IgG_2a_mAb served as isotype controls. Mouse anti-mouse CEACAM1 mAb CC1, rat anti-mouse CEACAM1 AgB10 [24] and rat anti-mouse E-cadherin and anti-mouse EpCAM mAbs were a kind gift from K. Holmes, University of Colorado, B. B. Singer, Charité Berlin, and P. Ruf, Trion Research, Munich, respectively. The cross-reactive mouse anti-human CEACAM mAb 4/3/17 (specific for human CEA/CEACAM5 in the mouse) were purchased from GENOVAC (Freiburg, Germany). Murine antibodies were detected with PE-conjugated goat anti-mouse IgG, rat antibodies with FITC-conjugated donkey anti-rat-IgG (DAKO).

### Detection of CEA and SV40 Tag by Western blotting

Exponentially growing gastric carcinoma cells, Cos7L cells, Cos7L-CEA transfectants, Meth-A cells and Meth-A-cTag transfectants which express a truncated cytoplasmically located SV40 Tag were harvested by trypsinization. Cells were washed three times in PBS and lysed at a density of 10^6 ^cells/ml in lysis buffer. Protein concentration was determined using the BCA Protein Assay Kit (Pierce, Rockford, Illinois, USA). Cell extracts corresponding to 10 μg of protein were separated by electrophoresis through 10% SDS polyacrylamide gels (Invitrogen, Karlsruhe, Germany), transferred to polyvinyliden fluoride membranes and incubated with 10 μg/ml anti-human CEACAM mAb 4/3/17 or a 1:100 diluted hamster anti-SV40 Tag antiserum (a kind gift by K.-H. Scheidtmann, University of Bonn). Bound antibodies were reacted with horse-radish peroxidase-labeled secondary antibodies and visualized using a chemiluminescence-based detection system (ECL; Amersham Biosciences Europe GmbH, Freiburg, Germany).

### Cell doubling time determination

*In vitro *doubling times of the cell lines were determined by plating the gastric carcinoma cells in 24-well plates in TM at the indicated starting cell numbers and counting of the cell samples from triplicate wells after gentle trypsinization every 3 days for 21 days. The *in vivo *doubling times were calculated from tumor volume measurements (see below) after inoculation with three different starting cell numbers (three mice per group). Doubling times were calculated from the log phase of the growth curves.

### Tumorigenicity and immunogenicity of the cell lines

For tumorigenicity assessment tumor cells were washed three times in PBS and 50 μl of cell suspensions with the indicated cell numbers were injected subcutaneously into the shaved right flank of the mice. To determine the immunogenicity, 10^7 ^tumor cells/ml were lysed by two consecutive freeze and thaw cycles. Mice were immunized four times at weekly intervals with 10^6 ^lysed tumor cells into the right flank. Three weeks later, the mice were challenged by subcutaneous injection of 3 × 10^6 ^viable tumor cells into the left flank. Experimental groups consisted of 4–6 mice. Tumor development was followed by serial measurements of the tumor size and the tumor volume was calculated according to the equation: tumor volume (mm^3^) = d^2 ^× D/2, where d and D were the shortest and the longest tumor diameter, respectively. Animals were euthanized when the tumors reached a volume of 300 mm^3^.

### Generation of Tag-specific cytotoxic T lymphocytes (CTL) and stimulation by gastric carcinoma cell lines

CTLs were generated as previously described[23]. Briefly, mice were intradermally inoculated with 1 μm gold particles coated with a Tag expression plasmid (BMG/cT-Ag.1 [23]) into the shaved abdominal skin using a helium pressure (200 psi) powered biolistic device (Helios gene gun; Bio-Rad Laboratories GmbH, Munich, Germany). Spleen cells obtained 14 days post vaccination were restimulated at weekly intervals with irradiated RBL5/T transfectants in RPMI-1640/10% FCS supplemented with 30 IU/ml IL-2. RBL5/T is a Rauscher virus-transformed T-lymphoma cell line derived from a C57BL6 (H-2b) mouse transfected with a SV40 Tag expression plasmid. To generate epitope-specific CTL, spleen cells taken 10 days post vaccination were restimulated in vitro with irradiated, Tag peptide-pulsed RBL5 cells. The T1, T2/3 and T4 epitope specificity of the CTL was controlled by determination of the IFNγ content of media upon stimulation with peptide-pulsed target cells using ELISA.

### Cytokine detection by ELISA

For capture and detection of IFNγ in supernatants by conventional sandwich ELISA, we used mAb R4-6A2 and biotinylated mAb XMG1.2, respectively (BD Pharmingen). Extinction was analyzed at 405/490 nm on a TECAN microplate ELISA reader (TECAN Crailsheim, Germany) with the EasyWin software (TECAN). The detection limit of the ELISA for IFNγ was 20 pg/ml.

## Results

### Establishment and phenotype of the gastric carcinoma cell lines

**Figure 1:**
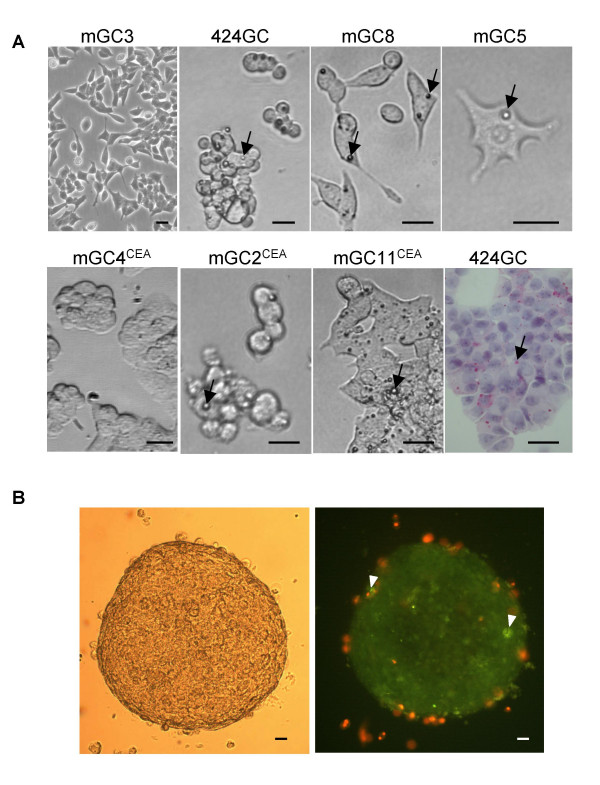
**Morphology of gastric carcinoma cell lines grown as monolayer cultures or as three dimensional spheroids**. (A) The cell lines show a slightly different epithelial morphology. Most cells of all cell lines contain a characteristic intracellular vacuole (arrows) which probably contains mucinous material stained red by the PAS method (last picture right in lower panel). (B) Spheroid formed by culturing mGC8 tumor cells on soft agar: left, phase contrast; right, fluorescence staining of necrotic cells with propidium iodide (red) and apoptotic cells with FITC-labeled annexinV (green, marked by arrowheads) as described in "Material and Methods". Magnification: bars correspond to 10 μm. mGC, murine gastric carcinoma.

**Figure 2:**
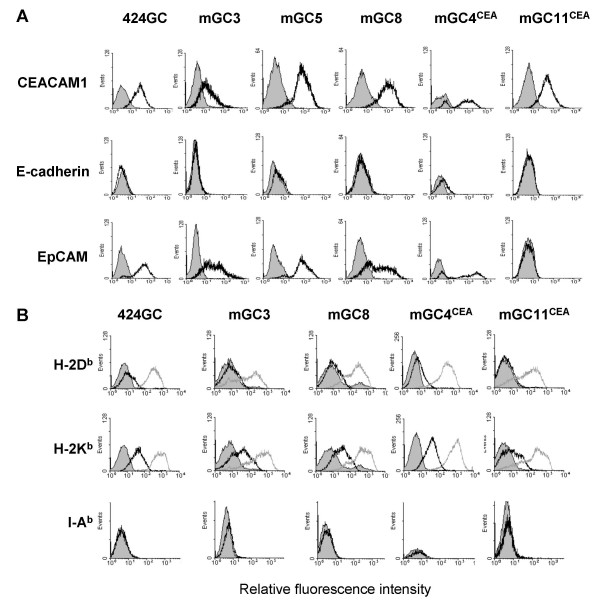
**Cell surface expression of epithelial markers (A) and MHC class I and II molecules (B)**. Gastric carcinoma cell lines were reacted either with PE-labeled (H-2K^b^, H-2D^b^, I-A^b^) or with unlabeled mAbs (CEACAM1, E-cadherin, EpCAM) followed by incubation with PE-labeled anti-mouse IgG or FITC-labeled anti-rat IgG and analyzed by flow cytometry. Histograms display the results obtained with antibodies against relevant antigens with (open gray) or without (open black) prior IFNγ stimulation and irrelevant antigens (gray filled curves).

From 8 gastric carcinoma samples 7 cell lines could be established. Four cell lines were derived from CEA424/Tag-transgenic mice (424GC, from a male mouse; mGC3, female; mGC5, male; and mGC8, female) and three lines from CEA424/Tag-CEA-transgenic mice (mGC2^CEA^, male; mGC4^CEA^, male; mGC11^CEA^, female). The time needed to obtain pure epithelial cell cultures varied greatly (mean 6 months, range 3–16 months). Although the tumor cells grow as adherent cells in culture, they tend to form aggregates rather than spreading over the culture substrate (Fig. 1A). The epithelial origin of the tumor cells was proven by morphological criteria including the presence of mucin within the cells (Fig. 1A) and the expression analysis of protein commonly expressed in epithelial cells (EpCAM, E-cadherin, CEACAM1) (Fig. 2A). A reduced content of mucin was found in all cell lines compared with the content in normal gastric epithelial cells but similar to that found in tumor cells within the gastric carcinoma in transgenic mice (Fig. 1A and [13]). All cell lines displayed CEACAM1 and EpCAM on their surface except mGC11^CEA^, none of them expressed E-cadherin (Fig. 2A and data not shown). All cell lines expressed MHC class I H-2K and, and at a much lower level, H-2D molecules (Fig. 2B). Expression of both proteins was strongly enhanced by IFNγ stimulation. No expression of MHC class II molecules (I-Ab) was detected (Fig. 2B and data not shown). CD54, CD80, CD86 or CD95 were not detected on any cell line (data not shown).

In order to determine the potential of the cell lines to be used for the generation of three-dimensional tumor models we analyzed tumor cell spheroid formation in vitro. As illustrated in Fig. 1B the cell lines formed compact tumor cell spheroids after 8 days of culture when 103 cells were seeded into soft agar-coated 96-well plates. Only minor intra-experimental variation was observed concerning the size of the spheroids. Staining with propidium iodide and FITC-labeled annexin V demonstrated that at least the surface layer of the spheroids consisted of viable cells with very few dead cells attached to it (Fig. 1B).

### Transgene expression by the gastric carcinoma cell lines

**Figure 3:**
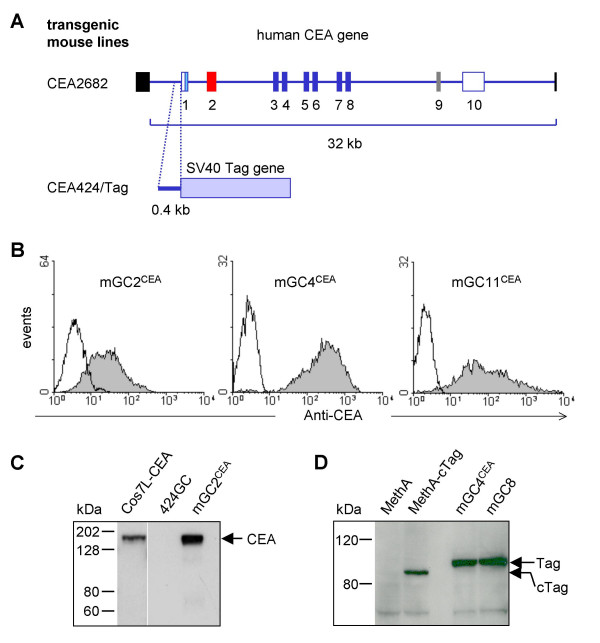
**Expression of CEA and Tag by gastric carcinoma cell lines derived from CEA424/Tag- or CEA424/Tag × CEA-transgenic mice**. (A) Structure of the CEA and CEA424/Tag transgenes. The exons 1–10 of the human CEA gene contained within the insert of cosmid clone cosCEA1[14] are shown as color coded boxes (light blue, leader; red, IgV-like domain; blue IgC-like domain; gray, transmembrane domain; white, 5' and 3'-untranslated region exons. Flanking vector sequences are indicated as black boxes. The location of the CEA minimal promoter present in the SV40 Tag gene transgene is indicated by dotted lines, the names of the transgenic lines are shown in the left margin. (B) Flow cytometry was performed by labeling of the indicated cells either with the CEA-specific mAb 26/3/13 (filled curves) or an isotype-matched antibody (open curves) followed by PE-labeled goat anti-mouse IgG antibodies. (C, D) For Western analysis, 10 μg of total protein from extracts of 424GC or mGC8 cells established from CEA424/Tag-transgenic mice and mGC2^CEA ^and mGC4^CEA ^cells from CEA424/Tag × CEA-transgenic mice were size separated by SDS polyacrylamide gel electrophoresis, transferred to a membrane and reacted with the CEA-specific mAb 26/3/13 (C) or hamster polyclonal anti-Tag antibodies (D). Extracts from Cos7L-CEA and Meth-A cells stably transfected with expression vectors encoding CEA or a Tag lacking a region with the nuclear localization signal (cTag) served as a positive control. The sizes of protein markers are indicated in the left margins.

**Figure 4:**
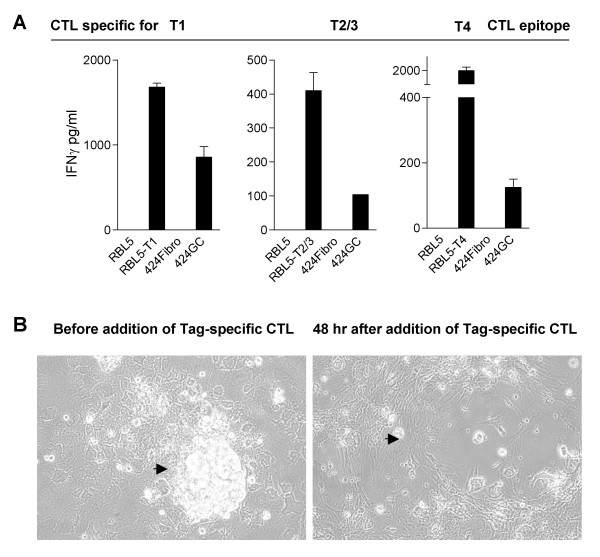
**MHC class I-restricted presentation of Tag epitopes by murine gastric carcinoma cell lines**. (A) Epitope-specific CTL generated in C57BL/6 mice by DNA immunization with a Tag expression vector and subsequent expansion by in vitro stimulation with Tag peptide-loaded RBL5 cells were incubated with irradiated 424GC, 424 fibroblasts, RBL5 and Tag T1, T2/3 or T4 peptide-pulsed RBL5 cells for 24 h and their IFNγ secretion into the culture media was determined by ELISA. Secretion of IFNγ by CTL stimulated with 424GC cells indicates that these cells present SV40Tag-specific peptides in an MHCI-restricted manner. (B) Coculture of 424GC and 424 fibroblasts were treated with 107 Tag-specific CTL in a petri dish for 48 h. Thereafter, non-adherent (dead) cells were removed. Left, coculture before CTL treatment; right, coculture after treatment. Arrows indicate the position of the tumor cells before addition of CTL.

CEA and Tag can serve as tumor-specific antigens (TSA) or tumor-associated antigens (TAA) after transplantation of the newly established tumor cell lines in immunocompetent syngeneic C57BL/6 and CEA- or Tag-transgenic mice, respectively. Although instrumental in tumor formation, Tag transgene expression is not always found in tumor cell lines derived from Tag-transgenic mice, like in TRAMP tumor cell lines[25]. This prompted us to analyze the expression and MHC class I-restricted presentation of the Tag transgene as well as the expression of CEA in the established gastric carcinoma cell lines. CEA cell surface expression was analyzed in cell lines derived from gastric tumors from double transgenic mice by flow cytometry and Western blot analysis. While the expression of the CEA transgene is regulated by the complete promoter region of the human CEA gene the expression of the Tag is controlled by a minimal -424/-8 bp CEA promoter (Fig. 3A). All three double-transgenic cell lines expressed CEA on the cell surface (Fig. 3B). In the gastric carcinoma cell line mGC2^CEA ^transgene-expressed CEA exhibited a molecular weight of 180 kDa similar to the one found in SV40-transformed African green monkey kidney cells stably transfected with a CEA expression vector and to CEA found in humans. As expected, no CEA was detected in 424GC cells, which were established from a CEA-negative Tag-transgenic mouse (Fig. 3C). Tag was found to be expressed by Western blotting and immunofluorescence analysis in all cell lines derived from single and double-transgenic mice (Fig. 3D and data not shown). This finding also supports the origin of the cell lines from the stomach carcinomas. Furthermore, the 424GC cell line efficiently presented endogenously processed Tag-derived peptides, especially the T1 epitope, in a MHC-I restricted manner as determined by the induction of IFNγ secretion byTag-specific CTL (Fig. 4A). Furthermore, the cell lines were efficiently killed by Tag-specific CTL in cytotoxic assays (Fig. 4B).

### Tumorigenicity of the gastric carcinoma cell lines

**Figure 5:**
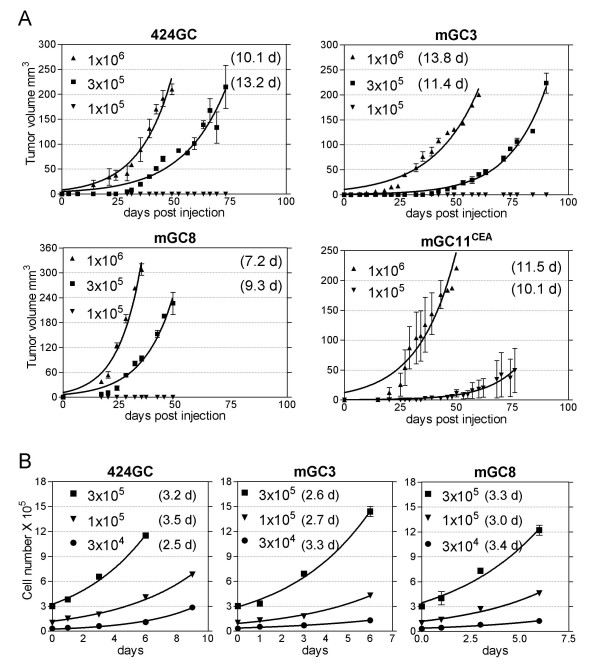
**In vivo (A) and in vitro growth characteristics (B) of murine gastric carcinoma cell lines**. Three mice each were injected with the indicated tumor cell doses. Tumor growth was quantified by two perpendicular measurements of the tumor diameter and calculation of the volume as described in the Materials and Methods section. To determine in vitro growth characteristics, cells were grown in 24-well plates starting with the indicated cell numbers. At different time points cells from triplicate wells were harvested and counted. Results are shown as mean +/- standard deviation (SD). Best fit curves as well as doubling times in days (shown in brackets) were calculated using the GraphPad software.

**Figure 6:**
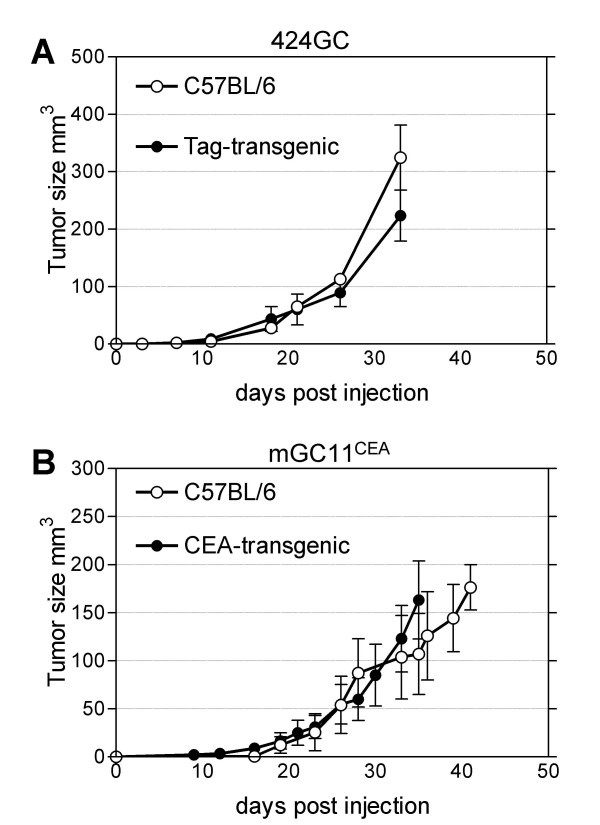
**Growth of gastric carcinoma cell lines in wild-type and transgenic mice**. 3 × 105 424GC cells were injected subcutaneously into C57BL/6 and CEA424/Tag-transgenic mice (A) or 3 × 105 mGC11^CEA ^cells were injected into C57BL/6 and CEA424/Tag × CEA-double transgenic mice (B). Tumor growth was quantified by two perpendicular measurements of the tumor diameter and calculation of the volume as described in the Materials and Methods section. Results are shown as mean +/- SD (n = 3).

To determine the tumorigenicity of the cell lines, various numbers (1 × 10^5^; 3 × 10^5^; 1 × 10^6^) of cells were injected subcutaneously into C57BL/6 mice. All cell lines were able to form tumors in 100% of the animals, if at least 3 × 10^5 ^tumor cells were injected (Fig. 5A). The tumors grew nearly exponentially without any delay until they reached a volume of 300 mm^3^. No distant metastases could be detected during the observation time. Western blot analysis performed on transplanted tumors demonstrated that both transgenes were expressed by the cell lines *in vivo *(data not shown). The doubling times of the tumor cells *in vivo *at a starting tumor load of 10^6 ^cells varied between 7.2 (mGC8) and 13.8 days (mGC3) (Fig. 5A). *In vitro*, all cell lines exhibited similar doubling times of about 3 days (Fig. 5B). We further compared subcutaneous tumor formation of the cell lines in wild-type (C57BL/6) and transgenic mice. No significant differences were observed in tumor take and tumor growth for the cell line 424GC when injected subcutaneously into wild-type or CEA424/Tag-transgenic mice (Fig. 6A) and for mGC11^CEA ^cells after injection into wild-type and CEA424/Tag-CEA-transgenic mice (Fig. 6B).

### Immunogenicity of the gastric carcinoma cell lines

**Figure 7:**
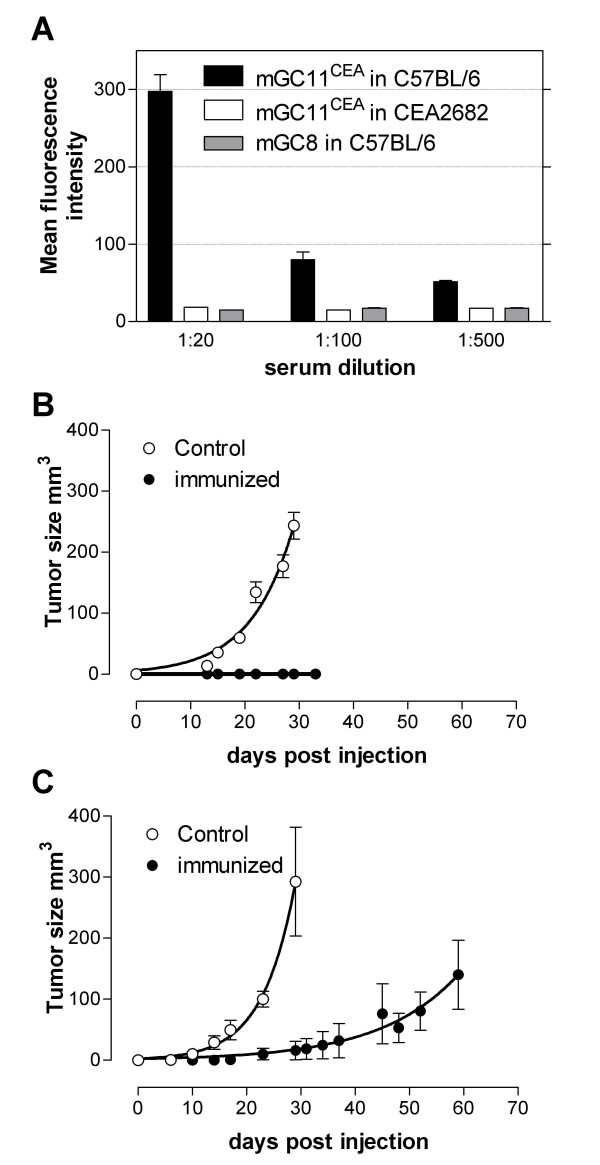
**Immunogenicity of mGC cells**. (A) Anti-CEA antibodies were determined in the serum of C57BL/6 and CEA-transgenic mice using flow cytometry. All mice had progressively growing tumors without overt necrosis after transplantation and growth of the indicated cell lines for 35–40 days. mGC4^CEA ^cells were incubated with the serum of the indicated mice at different dilutions and bound primary antibodies were detected with a PE-conjugated anti-mouse antibody. (B, C) Three C57BL/6 mice each were injected subcutaneously three times at weekly intervals with 1 × 106 mGC8 cells killed by two freeze-thaw cycles. Two weeks after the last vaccination, the mice were challenged by injection with 1 × 106 (B) and 3 × 106 (C) live mGC8 cells, respectively (filled-in circles). As a control, tumor cells were injected in non-immunized mice (open circles). Tumor volumes were calculated as described in Material and Methods section. Results are shown as mean values +/- SD.

The similar growth of the tumor cell lines in wild-type and transgenic mice indicates that no significant immune response to either tumor antigen (Tag, CEA) occurred in tumor bearing mice. Indeed, no Tag-specific CTL could be identified in the spleen of tumor bearing wild-type mice upon progressive subcutaneous growth of Tag-expressing gastric carcinoma cells (data not shown). However, when double transgenic cell lines grew in mice, CEA-specific antibodies could be identified in wild-type C57BL/6 mice but not in CEA-transgenic mice (Fig. 7A). Furthermore, three immunizations of C57BL/6 mice with 106 freeze-thawed mGC8 tumor cells at weekly intervals either prevented growth of subcutaneously injected live mGC8 cells completely or tumor outgrowth was delayed for nearly three weeks depending on the injected tumor cell dose (Fig. 7B, C). These experiments demonstrate that the tumor cell lines are immunogenic under certain conditions. However, C57BL/6 mice do not spontaneously mount an efficient tumor progression-limiting immune response to either tumor antigen during subcutaneous tumor growth.

## Discussion

The main goal of the present study was to establish a therapeutic model of gastric cancer in immunocompetent mice that would provide an animal model to evaluate anti-tumor immunity and immunotherapeutic strategies. Towards this end, the murine cell lines described in this paper were established from spontaneously developing gastric tumors of two different transgenic mouse strains. Together with the transgenic mice, these cell lines now provide a useful experimental system for gastric cancer. A similar experimental system for prostate cancer, the TRAMP mice in combination with the C1 and C2 cell lines has already proven its great impact on prostate cancer research [25-27]. This is of particular importance, since in contrast to the large number of human gastric carcinoma cell lines to our knowledge only one murine gastric carcinoma cell line is available. This cell line was established from the forestomach but not from the glandular part of the stomach from which the most common carcinomas arise in humans[28].

The cell lines which we have established from CEA424/Tag-transgenic and CEA424/Tag-CEA-transgenic mice, have most likely originated from transformed gastric glandular epithelial cells for several reasons: all cell lines have an epithelial phenotype, contain reduced but detectable amounts of mucin, and express, with one exception (mGC11^CEA^), the epithelial cell marker EpCAM. The mGC11^CEA ^cell line which was established from a CEA424/Tag-CEA-double transgenic mouse which expresses CEA, a molecule that is exclusively expressed by epithelial cells. Finally, all cell lines are tumorigenic and form tumors readily when transplanted subcutaneously into syngeneic mice (Fig. 5).

The murine gastric carcinoma cell lines as well as the primary gastric carcinomas mimic closely human gastric carcinomas and derived cell lines in expressing CEA (when established from tumor-bearing CEA-transgenic mice)[29], upregulate CEACAM1[30], downregulate E-cadherin expression[31] and express EpCAM[32]. CEA has been found to increase tumorigenicity by blocking differentiation[33], inhibiting anoikis, a mechanism which results in destruction of epithelial cells once detached from their basement membrane[34] and disrupting cell polarity[35]. CEACAM1 is downregulated in colonic, prostate and mammary carcinomas and its re-expression reduces the tumorigenicity of epithelial tumor cell lines[36,37]. Therefore, CEACAM1 is considered a tumor suppressor. On the other hand, CEACAM1 expression appears to be upregulated in subsets of other tumors like in stomach carcinomas in humans and mice[29,30]. It was shown for melanoma and lung adenocarcinomas, that CEACAM1 upregulation is significantly associated with a poor prognosis[30,38-40]. Furthermore, EpCAM has a direct effect on tumor cell proliferation by upregulation of c-myc and cyclin A/E[32] and loss of E-cadherin expression is observed in undifferentiated-type gastric carcinomas upon mucosal spread and deep invasion beyond the submucosa[31]. Taken together, these findings imply that similar molecular mechanisms are responsible for the tumorigenic properties of the murine gastric carcinoma cell lines as found for human gastric carcinomas and derived cell lines.

**Figure 8:**
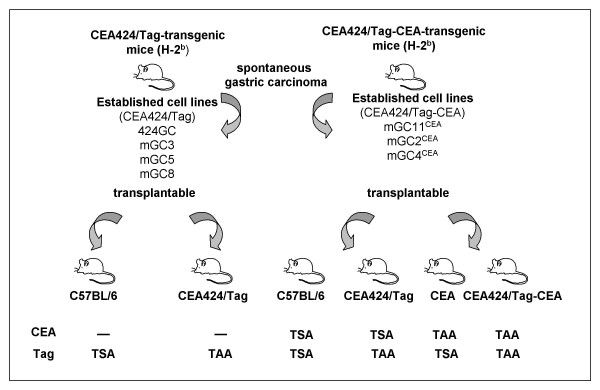
**Possible combinations of gastric tumor cell lines and mouse strains to generate mouse models which differ in the quality of the expressed tumor antigens and the anatomical location of the tumors**. The quality of the potential tumor antigens Tag and CEA can be manipulated by ectopic transplantation of Tag and/or CEA-positive tumor cell lines in wild-type C57BL/6 mice, Tag- or Tag × CEA-transgenic mice. Depending on whether the proteins represent self or foreign antigens they can be regarded as TAA or TSA. In spontaneously developing autochthonous tumors both CEA and Tag represent TAA.

Although spontaneously developing and autochthonously growing tumors are clinically the most relevant tumor models, in many cases transplantable tumors are needed to create a large body of data from multiple treatment regimens. From an economic point of view it is reasonable to test only the most promising strategies in the more challenging transgenic mouse models. In this context it is advantageous that the Tag and CEA-expressing gastric carcinoma cell lines are growing equally well in transgenic and wild-type C57BL/6 mice despite their different immunogenicity in both hosts. This permits the stepwise optimization of therapy strategies in models gradually approaching the patients' situation with respect to tumor antigen tolerance. Therefore, in wild-type mice Tag and CEA can serve as TSA, which could be used as target antigens in different immunotherapy approaches. In this particular setting, many immunotherapies should be effective to a certain extent, which is a prerequisite for the optimization of the therapy protocol. Indeed, we could demonstrate in the present report that simple immunization with tumor cell lysates is able to induce protective anti-tumor immunity (Fig. 7). Once the therapy protocol has been optimized for the wild-type model, the intricacy of the model can be increased, e.g. by transplanting the tumor cells into transgenic mice. In mice expressing only one of the two potential tumor antigens, the antigens would either function as TSA or TAA, whereas in CEA424/Tag-CEA double-transgenic mice both antigens would represent TAA (Fig. 8). In these different models, further rounds of therapy optimization can be performed. This is expected to lead to an optimal immunotherapy protocol effective in transgenic mice suffering from spontaneously developing and autochthonously growing tumors which represent the most relevant model for human cancer. In this context, the consistent expression of the widely used and well-characterized tumor antigens Tag and CEA as well as high level (inducible) expression of MHC class I molecules needed for antigen presentation to cytotoxic T cells are instrumental for the use of the cell lines to study antigen-specific tumor immune therapies. Indeed, we could demonstrate that the level of Tag expression of the 424GC cell line is sufficient to be killed by syngeneic Tag-specific CTL. In contrast, other Tag-based transgenic mouse models where primary tumor-derived cell lines exist, like the TRAMP model (cell lines C1, C2 and C3), have been reported to lack Tag expression in derived cell lines[25].

It is of particular relevance that the gastric cancer cell lines express different potential target antigens since the type of immunological tolerance may differ between different tumor antigens and between different transgenic strains. Indeed it was shown that depending on the expression pattern of Tag in different Tag-transgenic mice one can encounter all facets of immunological tolerance, ranging from immunological ignorance to central tolerance[25,41-43]. There is strong evidence that also in CEA424/Tag mice central tolerance against the Tag exists (R. K., and W. Z., unpublished results). On the other hand, only a peripheral tolerance appears to exist for CEA in CEA2682-mice [44-47]. If needed, this allows testing of strategies to break tolerance to CEA without interference of the viral antigen Tag.

Finally, the cell lines generated from the transgenic mice have significant advantages to other subcutaneously transplantable CEA-expressing murine tumor cell lines, since in the transgenic cell lines CEA is expressed under the control of its naturally existing regulatory elements, which may be relevant in respect to possible immune escape mechanisms (i.e. escape variants).

## Conclusion

For the optimization of tumor immunotherapy protocols, a series of *in vivo *models are needed which gradually make higher demands on the efficacy of the therapy. Ideally the whole series should be composed of the same cellular system and reach high relevance for the human disease for which immunotherapy is to be developed. The model system composed of wild-type C57BL/6, CEA424/Tag, CEA2682 and CEA424/Tag-CEA transgenic mice and the respective tumor cell lines described in the present report offers such a model for immunotherapies of gastric carcinoma. The established murine gastric adenocarcinoma cell lines may also be helpful for the identification of new tumor antigens relevant to gastric cancer as well as for the understanding of molecular events during gastric cancer progression.

## Abbreviations

CEA, carcinoembryonic antigen; GC, gastric carcinoma; TAA, tumor-associated antigen; Tag, SV40 virus large T antigen; TSA, tumor-specific antigen.

## Competing interests

The author(s) declare that they have no competing interests.

## Authors' contributions

JN and NvdE together collected most of the data, carried out animal experiments and participated in drafting the manuscript. RAH participated in designing the study and revising critically the manuscript. HW designed animal experiments and interpreted the data. WZ co-designed the study and participated in manuscript writing. RK conceived and coordinated the study, established cell lines and participated in preparing the manuscript. All authors read and approved the final manuscript.

## Pre-publication history

The pre-publication history for this paper can be accessed here:
